# Financial difficulties experienced by patients with gastrointestinal stromal tumours (GIST) in the Netherlands: data from a cross-sectional multicentre study

**DOI:** 10.1007/s00520-024-08451-0

**Published:** 2024-04-10

**Authors:** Deborah van de Wal, Dide den Hollander, Ingrid M. E. Desar, Hans Gelderblom, Astrid W. Oosten, Anna K. L. Reyners, Neeltje Steeghs, Olga Husson, Winette T. A. van der Graaf

**Affiliations:** 1 Department of Medical Oncology, The Netherlands Cancer Institute, Amsterdam, The Netherlands; 2 Department of Medical Oncology, Radboud University Medical Centre, Nijmegen, The Netherlands; 3 Department of Medical Oncology, Leiden University Medical Centre, Leiden, The Netherlands; 4 Department of Medical Oncology, Erasmus MC Cancer Institute, Erasmus University Medical Centre, Rotterdam, The Netherlands; 5 Department of Medical Oncology, University Medical Centre Groningen, University of Groningen, Groningen, The Netherlands; 6 Department of Clinical Pharmacology, The Netherlands Cancer Institute, Amsterdam, The Netherlands; 7 Department of Psychosocial Research and Epidemiology, The Netherlands Cancer Institute, Amsterdam, The Netherlands; 8 Department of Surgical Oncology, Erasmus MC Cancer Institute, Erasmus University Medical Centre, Rotterdam, The Netherlands

**Keywords:** Financial difficulties, Financial toxicity, Patient-reported outcome, Quality of life, Gastrointestinal stromal tumour, Tyrosine kinase inhibitors

## Abstract

**Purpose:**

This study aims to (1) explore the prevalence of patient-reported financial difficulties among GIST patients, differentiating between those currently undergoing tyrosine kinase inhibitor (TKI) treatment and those who are not; (2) investigate associations between financial difficulties and sociodemographic and clinical characteristics, work, cancer-related concerns, anxiety and depression and (3) study the impact of financial difficulties on health-related quality of life.

**Methods:**

A cross-sectional study was conducted among Dutch GIST patients diagnosed between 2008 and 2018, who were invited to complete a one-time survey between September 2020 and June 2021. Patients completed nine items of the EORTC item bank regarding financial difficulties, seven work-related questions, the Hospital Anxiety and Depression Scale, Cancer Worry Scale and EORTC QLQ-C30.

**Results:**

In total, 328 GIST patients participated (response rate 63.0%), of which 110 (33.8%) were on TKI treatment. Patients currently treated with TKIs reported significantly more financial difficulties compared to patients not on TKIs (17.3% vs 8.7%, *p* = 0.03). The odds of experiencing financial difficulties was 18.9 (95% CI 1.7–214.7, *p* = 0.02) times higher in patients who were less able to work due to their GIST diagnosis. Patients who experienced financial difficulties had significantly lower global quality of life and functioning, and more frequently reported psychological symptoms as compared to patients who did not report financial difficulties.

**Conclusion:**

Even in a country where the costs of TKIs and follow-up care are covered by health insurance, financial difficulties can be present in GIST patients, especially in patients on TKI treatment, and may negatively influence the quality of life.

**Supplementary Information:**

The online version contains supplementary material available at 10.1007/s00520-024-08451-0.

## Introduction

Financial difficulty, also described as financial toxicity, financial distress or financial burden, is an increasing topic of interest in cancer care [1]. It describes the financial burden and distress that can arise for patients, and their family members, as a result of their cancer diagnosis and treatment [2, 3]. Financial toxicity results from both objective financial burden due to the direct and indirect costs of cancer treatment, which increase over time from diagnosis, and subjective distress [4]. Financial difficulties can be influenced by multiple factors such as disease and treatment-related factors (i.e. type of cancer, its severity, type of treatment), health insurance coverage, additional disease and treatment-related cost that are not covered (e.g. travel and parking costs for the patient and relatives) and personal circumstances (i.e. whether a patient is the wage earner in the family, has savings, and whether a patient or family member can continue work) [5]. Also, in long-term cancer survivors, unemployment and limited financial resources were found to be risk factors for developing financial toxicity [6]. Experiencing financial difficulties can subsequently diminish the quality of life; increase symptoms of anxiety, stress, and depression; lead to isolation; reduce adherence to treatment; limit access to care and lead to a greater risk of mortality [7–11].

Financial toxicity might be more present in patients diagnosed with a rare cancer, such as sarcomas. It could be hypothesized that these patients experience more financial difficulties than patient with common cancers due to their treatment and follow-up in expertise centres, with often greater travel distances, and a disease that is less well known and more unpredictable than common cancers which make a general judgement on options of, for example, return to work more complex than in patients with common cancers. Financial toxicity has been studied in a large group of 1103 sarcoma patients, in which nearly half of the patients reported financial toxicity, which was associated with receiving a disability pension, being currently on sick leave, and having a disability parking pass [12]. This study also included 130 gastrointestinal stromal tumour (GIST) patients of whom 39% reported financial toxicity. GISTs are a unique group within sarcomas, as the treatment (e.g. tyrosine kinase inhibitors (TKIs)) is different as well as the life expectancy of patients (e.g. over 5 years on average in metastatic setting). Therefore, it is of interest to analyse GIST patients as a separate group. GISTs are rare tumours with an incidence of approximately 8 per million person‐years in the Netherlands [13]. The mainstay of treatment for localised GISTs is complete surgical resection with neo-adjuvant imatinib considered for larger tumours and where significant morbidity of surgery is anticipated. In patients with high-risk disease, based on the tumour size, localisation and mitotic rate, adjuvant imatinib for 3 years is recommended after surgical resection [14]. Furthermore, one in five patients present with metastatic disease at diagnosis [1], while others may develop metastases during follow-up. Metastatic GIST patients depend on life-prolonging treatment with TKIs, such as imatinib, which significantly improved their median overall survival from 12 to 68 months [15].

In our previous qualitative study, financial difficulties were already acknowledged by GIST survivors on long-term TKI treatment [16]. Their financial difficulties were often a result of loss of income, which coincided with concerns about being able to pay for everything. In most cases, a reduced income was work-related, patients expressed not being able to work full-time, having to change jobs or losing their job. Besides that, patients underlined their inability to buy a house due to difficulties with insurance and mortgages, and patients reported higher expenses on health insurance because of being chronically ill. In general, financial difficulties as a result of illness and treatment get little attention in daily clinical practice [17], while they can have a considerable impact on both the patient’s quality of life and treatment outcomes [18, 19]. To gain more insight into financial difficulties among GIST patients, we conducted a cross-sectional study in the Netherlands with the aim to (1) explore the prevalence of financial difficulties among GIST patients, differentiating between those on tyrosine kinase inhibitor (TKI) treatment and those who are not; (2) investigate associations between financial difficulties and sociodemographic and clinical characteristics, work, cancer-related concerns, anxiety and depression; and (3) study the impact of financial difficulties on HRQoL.

## Methods

### Study design and data collection

Data of the cross-sectional ‘Life with GIST’ study was used, which was approved by the medical ethical committee of the Radboud University Medical Centre (2019–5888). This study was conducted among adult (≥ 18 years) GIST patients diagnosed between January 2008 and December 2018, registered in the Netherlands Cancer Registry (NCR) and treated within one of the GIST expert centres (Radboud University Medical Centre [Nijmegen], Erasmus Medical Centre [Rotterdam], Leiden University Medical Centre, Netherlands Cancer Institute [Amsterdam] and University Medical Centre Groningen). Patients were not eligible when they had a cognitive impairment, were not able to read and understand the Dutch language, or were too ill at the time of the study based on the advice of their (former) treating specialist. After patients provided informed consent, including permission to link their study data to data from the NCR, patients completed the survey online or on paper. Data were collected within Patient-Reported Outcomes Following Initial treatment and Long-term Evaluation of Survivorship (PROFILES) registry [20] from September 2020 through June 2021.

### Study measures

#### Sociodemographic and clinical characteristics

Sociodemographic data, co-morbidities, tumour and treatment characteristics were patient-reported. Additional data (e.g. socio-economic status) and missing clinical data were derived from the NCR database, if available.

#### Financial difficulties

Financial toxicity was assessed by nine items of the EORTC item bank [21] regarding financial difficulties caused by the patient’s physical condition or treatment. All items of the EORTC item library are systematically developed, tested and validated [22]. Items were scored on a 4-point Likert scale ranging from 1 (not at all) to 4 (very much); for our analyses, items were scored as either present (score of 2–4) or not present (score of 1). To also reflect on the grade of financial difficulties, we calculated a mean score for all items. Prior to this, we performed a linear transformation, so scores ranged from 0 to 100, with a higher score indicating a higher burden.

#### Work and workability index

The survey included questions about having a paid job and reasons for not having a paid job. The ability to perform work was measured with three single items extracted from the workability index (WAI) [23], including (1) Is your work psychologically, physically or physically and psychologically demanding?, (2) How many points (1–10) would you give your current work ability compared to highest workability ever? (3) Is your illness a hindrance to your current job?

#### Cancer-related concerns

To assess cancer-related concerns, we used the Cancer Worry Scale (CWS) [24], which consists of eight items regarding concerns about cancer recurrence or progression. All items were scored on a 4-point Likert scale ranging from 1 (never) to 4 (almost always). Total CWS scores ranged from 8 to 32, with a score of 14 or higher being indicative of severe fear of cancer recurrence or progression [25].

#### GIST-specific concerns

Three separate items that we designed ourselves were used to assess GIST-specific concerns about the need for TKI treatment in the future, dying from GIST in the near future and dying from GIST in the long term future. These items were also scored on a 4-point Likert scale ranging from 1 (never) to 4 (almost always), and later categorized as absent (1) or present (2–4).

#### Symptoms of anxiety and depression

The Hospital Anxiety and Depression Scale (HADS) was used to assess symptoms of anxiety and depression [26]. This 14-item measure consists of 7 items on anxiety and 7 items on depression. Scores ranged from 0 to 21, with a score of 8 or higher indicating possible symptoms of anxiety or depression, respectively [27].

#### Health-related quality of life

To investigate the impact of financial difficulties on HRQoL, we used the EORTC QLQ-C30 [28], which incorporates a global quality of life scale and five functioning scales (i.e. physical, role, cognitive, emotional, and social). The item ‘physical condition or treatment causing financial difficulties’ was used to determine whether financial difficulties were present or not. All items of the QLQ-C30 were scored on a 4-point Likert scale ranging from 1 (not at all) to 4 (very much), except for the items regarding global health and quality of life which were scored from 1 (very poor) to 7 (excellent). A linear transformation was conducted to standardize the raw scores of the scales; hence, scores ranged from 0 to 100, with higher scores indicating a better quality of life for the global quality of life and functioning scales.

### Statistical analysis

All statistical analyses were carried out using SPSS Statistics (IBM Corporation, version 26.0, Armonk, NY, USA). Two-sided *p*-values of < 0.05 were considered statistically significant. All variables were described as means and standard deviations (continuous data) or frequencies and percentages (categorical data). Independent sample *t*-tests (continuous data) and chi-square tests (categorical data) were conducted to compare GIST patients on self-reported current TKI treatment and not on TKI treatment on sociodemographic and clinical variables, work-related variables and financial difficulty items. Univariable logistic regression analyses were performed to determine the association between financial difficulties and sociodemographic and clinical characteristics, work-related items, cancer-related concerns, GIST-specific concerns, and symptoms of anxiety and depression in the total population. Independent variables with *p* < 0.1 were included in the multivariable logistic regression analyses, after being tested for multi-collinearity using the variance inflation factors and variance proportions test.

The analyses in the main body of this manuscript are based on patient-reported and NCR data and are stratified for self-reported current TKI treatment. Differences were observed between what patients reported and what the NCR recorded, particularly on treatment setting (i.e. curative or palliative). To also perform sensitivity analysis on the most accurate data, a linkage with the Dutch GIST registry (DGR) was made, of which the results, stratified by treatment setting based on DGR data, are presented as supplementary material. The differences between both analyses are addressed in the results. The DGR is a prospectively maintained database, since 2009, of GIST patients treated in one of the five GIST expert centres in the Netherlands, approved by the local independent ethics committee (IRBd20-212). Patients consented to linkage with the DGR by signing the informed consent form.

## Results

A total of 521 GIST patients were invited, of whom 328 (response rate 63%) completed the survey, and 325 patients indicated whether or not they were on current TKI treatment. Patients had a mean age of 66.6 years at moment of the survey and were on average 5.9 years (range 1.7–12.6 years) after diagnosis. The majority of patients (*n* = 245, 76.1%) were married or lived with a partner and had a high socio-economic status (*n* = 178, 54.8%) and a low to intermediate educational level (*n* = 204, 63.9%). Of the 325 patients, 110 were currently treated with TKIs, mostly imatinib (*n* = 95, 86.4%). An overview of the patient characteristics is presented in Table 1.

**Table 1:** Patient characteristics stratified by self-reported current TKI treatment

	Total (*n* = 325)	Current TKI (*n* = 110)	Non-current TKI (*n* = 215)	*p*-value
Sex *n* (%)				
Male	173 (53.2)	60 (54.5)	113 (52.6)	0.73
Female	152 (46.8)	50 (45.5)	102 (47.4)	
Age at survey completion, mean ± SD	66.6 ± 10.4	66.6 ± 9.7	66.7 ± 10.7	0.90
Socio-economic status, *n* (%)				
Low	147 (45.2)	52 (47.3)	95 (44.2)	0.60
High	178 (54.8)	58 (52.7)	120 (55.8)	
Marital stage, *n* (%)				
Married/living with partner	245 (76.1)	86 (78.9)	159 (74.6)	0.40
Not living with a partner	77 (23.9)	23 (21.1)	54 (25.4)	
Missing	3	1	2	
Educational level*, *n* (%)				
Low/intermediate	204 (63.9)	69 (63.9)	135 (64.0)	0.99
High	115 (36.1)	39 (36.1)	76 (36.0)	
Missing	6	2	4	
Comorbidity, *n* (%)				
None	108 (33.4)	35 (32.1)	73 (34.1)	0.60
1	70 (21.7)	21 (19.3)	49 (22.9)	
≥ 2	145 (44.9)	53 (48.6)	92 (43.0)	
Missing	2	1	1	
Time since diagnosis in years, mean ± SD	5.9 ± 2.8	6.0 ± 2.8	5.8 ± 2.8	0.63
Location primary GIST, *n* (%)				
Stomach	205 (63.1)	53 (48.2)	152 (70.7)	**< 0.01**^a^
Small intestine	78 (24.0)	37 (33.6)	41 (19.1)	
Rectum	21 (6.5)	8 (7.3)	13 (6.0)	
Other	21 (6.5)	12 (10.9)	9 (4.1)	
Treatment setting				** < 0.01**
Curative setting	259 (80.2)	46 (42.2)	213 (99.5)	
Palliative setting	64 (19.8)	63 (57.8)	1 (0.5)	
Missing	2	1	1	
Received TKI at some point				
Yes	211 (65.3)	110 (100.0)	101 (47.4)	**< 0.01**
No	112 (34.7)	0	112 (52.6)	
Missing	2	-	2	
Received previous surgery for the GIST				
Yes	297 (92.0)	86 (78.2)	211 (99.1)	**< 0.01**^a^
No	26 (8.0)	24 (21.8)	2 (0.9)	
Missing	2	-	2	
Phase of treatment according to patient report				
Declared cured, no follow-up	61 (18.9)	-	61 (28.6)	**< 0.01**^a^
Not receiving active treatment, in follow up	151 (46.9)	-	151 (70.9)	
Receiving active treatment with curative intent	46 (14.3)	46 (42.2)	-	
Receiving active treatment with palliative intent	63 (19.6)	63 (57.8)	-	
Palliative intent without treatment	1 (0.3)	-	1 (0.5)	
Missing	3	1	2	

Abbreviations: *TKI* tyrosine kinase inhibitor, *SD* standard deviation

^*^Low (primary and secondary education), intermediate ((secondary) vocational education) and high (higher vocational education and academic education) educational level

^a^Fisher’s exact test or likelihood ratio

Data in bold emphasis indicates significant results

### Financial difficulties

Patients on TKIs reported significantly more financial difficulties due to their physical condition or treatment compared to patients not on TKIs (17.3% vs 8.7%, *p* = 0.03), and more often changed their lifestyle because of financial difficulties (14.5% vs 5.8%, *p* = 0.01). The most frequently reported financial difficulty in both groups entailed extra expenses as a result of the GIST and treatment, which was more present in patients on TKIs (40.9%) compared to those not on TKIs (32.5%), but this was not significantly different. In Table 2, the prevalence and means of the different financial difficulties are reported.

**Table 2:** Patient-reported financial difficulties as a result of the GIST or medical treatment of patients on current TKI and not on TKI

		Total (*n* = 316*)	Current TKI (*n* = 110)	Non-current TKI (*n* = 206)	***p***-value
Physical condition or treatment causing financial difficulties	Yes No	37 (11.7) 279 (88.3)	19 (17.3) 91 (82.7)	18 (8.7) 188 (91.3)	**0.03**
	Mean ± SD	5.8 ± 17.8	8.8 ± 22.0	4.2 ± 14.9	0.05
Having extra expenses that were difficult to pay	Yes No	27 (8.5) 289 (91.5)	13 (11.8) 97 (88.2)	14 (6.8) 192 (93.2)	0.14^a^
	Mean ± SD	3.3 ± 11.3	4.8 ± 14.2	2.4 ± 9.3	0.11
Having extra expenses	Yes No	112 (35.4) 204 (64.6)	45 (40.9) 65 (59.1)	67 (32.5) 140 (67.5)	0.14
	Mean ± SD	15.5 ± 23.0	18.5 ± 25.0	13.9 ± 21.9	0.11
Lacking money to buy basic things	Yes No	14 (4.4) 302 (95.6)	8 (7.3) 102 (92.7)	6 (2.9) 200 (97.1)	0.09^a^
	Mean ± SD	2.1 ± 10.4	3.6 ± 13.8	1.3 ± 8.0	0.10
Being in debt	Yes No	7 (2.2) 309 (97.8)	4 (3.6) 106 (96.4)	3 (1.5) 205 (98.5)	0.24^a^
	Mean ± SD	1.1 ± 7.4	2.1 ± 11.3	0.5 ± 4.0	0.15
Changing one's lifestyle because of financial difficulties	Yes No	28 (8.9) 288 (91.1)	16 (14.5) 94 (85.5)	12 (5.8) 194 (94.2)	**0.01**^a^
	Mean ± SD	4.6 ± 16.1	7.9 ± 21.1	2.9 ± 12.4	**0.03**
Having less money to spend on oneself	Yes No	29 (9.2) 287 (90.8)	12 (10.9) 98 (89.1)	17 (8.3) 191 (91.7)	0.42^a^
	Mean ± SD	4.6 ± 15.9	6.0 ± 19.3	3.9 ± 13.8	0.30
Experiencing problems paying regular expenses	Yes No	14 (4.4) 302 (95.6)	6 (5.5) 104 (94.5)	8 (3.9) 198 (96.1)	0.57^a^
	Mean ± SD	2.0 ± 9.9	2.7 ± 12.1	1.6 ± 8.6	0.34
Having to borrow money or sell personal belongings	Yes No	9 (2.9) 306 (97.1)	5 (4.5) 105 (95.5)	4 (2.0) 203 (98.0)	0.29^a^
	Mean ± SD	1.2 ± 7.2	1.8 ± 8.8	0.8 ± 6.1	0.24

Abbreviations: *TKI* tyrosine kinase inhibitor, *SD* standard deviation

^*^9 patients did not completed the items regarding financial difficulties

^a^Fisher’s exact test

Data in bold emphasis indicates significant results

### Work and workability

A covariate that can contribute to financial difficulties is employment. In our study, 219 (68.9%) patients indicated not having a paid job, in most cases due to retirement (82.8%). Ninety-nine (31.1%) patients had a paid job; this percentage was significantly (*p* = 0.04) lower in the group of patients on TKIs (*n* = 26, 24.3%) compared to those not on TKIs (*n* = 73, 34.6%). More detailed information about GIST patients and work is presented in Table 3.

**Table 3:** Patient-reported work-related information of GIST patients on TKI and not on TKI

**Patients with a paid job**	**Total** (*n* = 99*)	**Current TKI (*n* = 26)**	Non-current TKI (*n* = 73*)	***p*-value (0.04)**
Working hours**				0.17
Mean ± SD	32.1 ± 10.8	29.5 ± 11.7	33.0 ± 10.3	
Range	6–60	6–50	8–60	
Type of work				
Physical	8 (8.2)	4 (15.4)	4 (5.6)	0.24^a^
Mental	54 (55.7)	15 (57.7)	39 (54.9)	
Both physical and mental	35 (36.1)	7 (26.9)	28 (39.4)	
Able to work				
Not–less able	7 (7.2%)	4 (15.4)	3 (4.2)	0.08^a^
Good–excellent	90 (92.8%)	22 (84.6)	68 (95.8)	
Work is hindered by				
Nothing	56 (57.7)	12 (46.2)	44 (62.0)	0.17
Working causes me some complaints	13 (13.4)	5 (19.2)	8 (11.3)	0.33^a^
Have to reduce my work pace or adjust my way of working	21 (21.6)	8 (30.8)	13 (18.3)	0.27^a^
Often have to reduce my work pace or adjust my way of working	10 (10.3)	4 (15.4)	6 (8.5)	0.45^a^
Have the feeling that I can only work part-time	9 (9.3)	4 (15.4)	5 (7.0)	0.24^a^
I dare not to apply for another job	5 (5.2)	1 (3.8)	4 (5.6)	1.00^a^
I am not able to work at all	2 (2.1)	-	2 (2.8)	0.37^a^
**Patients not having a paid job**	**Total (*n* = 219)**	**Current TKI (*n* = 83)**	**Non-current TKI (*n* = 136)**	***p*-value (0.04)**
Reasons for not having a paid job				
Retired	178 (82.8)	57 (70.4)	121 (90.3)	**< 0.01**^a^
Unwillingly without a job	5 (2.3)	1 (1.2)	4 (3.0)	
Declared incapacitated	25 (11.6)	20 (24.7)	5 (3.7)	
Staying home wife/man, caregiver	7 (3.3)	3 (3.7)	4 (3.0)	
Missing	4	2	2	
Declared incapacitated due to cancer	22 (88.0)	19 (95.0)	3 (60.0)	**0.04**^a^
Declared incapacitated, for				
70–80%	5	3	2	
100%	18	16	2	
Missing	2	-	2	

Abbreviations: *TKI* tyrosine kinase inhibitor, *SD* standard deviation

^*^Two missing, these two patients indicated having a job but did not completed the work-related questions

^**^In the Netherlands, an average of 36 working hours is considered a full-time job

^a^Fisher’s exact test or likelihood ratio

Data in bold emphasis indicates significant results

### Factors associated with various financial difficulties

Our analysis showed that financial difficulties were not associated with socio-economic status, educational level or gender (Table 4). Being less able to work was associated with 18.9 (95% CI 1.7–214.7) higher odds of experiencing financial difficulties due to the GIST or treatment and 31.0 (95% CI 1.2–830.8) higher odds of changing one’s lifestyle because of financial difficulties. Patients concerned about the need for TKI treatment in the future had 1.7 (95% CI 1.1–2.9) higher odds of having extra expenses, and patients with severe fear of recurrence or progression had 9.7 (95% CI 1.1–89.0) higher odds of experiencing problems paying regular expenses. Having symptoms of anxiety was associated with 27.7 (95% CI 1.1–724.2) higher odds of having extra expenses that were difficult to pay, 17.6 (95% CI 1.4–221.9) higher odds of being in debt and 55.4 (95% CI 5.4–570.0) higher odds of having to borrow money or sell personal belongings. Not living with a partner was associated with 7.6 (95% CI 1.7–34.6) higher odds of lacking money to buy basic things, while previously received surgery for the GIST was associated with 0.1 (95% CI 0.0–0.8) lower odds of lacking money to buy basic things.

**Table 4:** Logistic regression models evaluating factors associated with various financial difficulties

**Physical condition or treatment causing financial difficulties**	**Univariable logistic regression**		**Multivariable logistic regression** Nagelkerke *R*^2^ = 0.34	
	**OR (95% CI)**	***p*-value**	**OR (95% CI)**	***p*-value**
Current TKI use	2.2 (1.1–4.4)	0.03	3.0 (0.5**–**16.8)	0.21
Time since GIST diagnosis (in years)	1.1 (1.0**–**1.3)	0.07	1.2 (0.9**–**1.5)	0.21
GIST location other than the stomach	2.9 (1.4**–**5.8)	< 0.01	0.8 (0.2**–**4.3)	0.81
Less able to work due to GIST and treatment	13.5 (2.6**–**71.3)	< 0.01	18.9 (1.7**–**214.7)	**0.02**
Declared incapacitated for work	4.2 (1.5**–**11.6)	0.01		
Symptoms of anxiety	5.5 (2.6**–**11.7)	< 0.01	5.3 (0.5**–**62.5)	0.18
Symptoms of depression	5.0 (2.3**–**11.1)	< 0.01	0.1 (0.0**–**2.6)	0.17
Severe fear of recurrence or progression	4.1 (1.9**–**9.0)	< 0.01	2.8 (0.4**–**18.2)	0.27
Concerned about the need for TKI treatment in the future	2.0 (1.0**–**4.0)	0.06	1.9 (0.4**–**9.8)	0.45
Concerned about dying from GIST in the near future	3.4 (1.7**–**7.1)	< 0.01	1.3 (0.2**–**10.3)	0.83
Concerned about dying from the GIST in the long term future	3.2 (1.4**–**7.02)	< 0.01	1.2 (0.1**–**9.9)	0.87
**Having extra expenses that were difficult to pay as a result of physical condition or medical treatment**	**Univariable logistic regression**		**Multivariable logistic regression** Nagelkerke *R*^2^ = 0.41	
	**OR (95% CI)**	***p*-value**	**OR (95% CI)**	***p*-value**
Not living with a partner	2.1 (.9**–**4.8)	0.08	1.9 (0.2**–**15.3)	0.56
Receiving treatment in a palliative setting	2.2 (.9**–**5.1)	0.08	0.6 (0.0**–**17.5)	0.79
Having ≥ 2 comorbidities	3.0 (1.1**–**8.2)	0.04	1.5 (0.2**–**12.0)	0.70
Less able to work due to GIST and treatment	4.7 (.8**–**28.7)	0.10	7.6 (0.5 – 117.6)	0.15
Declared incapacitated for work	3.8 (1.2**–**11.9)	0.03		
Symptoms of anxiety	7.5 (3.2**–**17.4)	< 0.01	27.7 (1.1**–**724.2)	**0.05**
Symptoms of depression	6.3 (2.7**–**15.0)	< 0.01	0.0 (0.0**–**2.3)	0.12
Severe fear of recurrence or progression	5.3 (2.1**–**13.4)	< 0.01	8.5 (0.6**–**116.5)	0.11
Concerned about the need for TKI treatment in the future	2.5 (1.1**–**5.8)	0.03	4.3 (0.5**–**36.0)	0.18
Concerned about dying from GIST in the near future	4.3 (1.8**–**10.2)	< 0.01	0.4 (0.0**–**4.1)	0.43
Concerned about dying from the GIST in the long term future	5.9 (2.0**–**17.4)	< 0.01	1.8 (0.1**–**21.3)	0.65
**Having extra expenses as a result of physical condition or medical treatment**	**Univariable logistic regression**		**Multivariable logistic regression** Nagelkerke *R*^2^ = 0.10	
	**OR (95% CI)**	***p*-value**	**OR (95% CI)**	***p*-value**
Age at moment of questionnaire (in years)	1.0 (1.0**–**1.0)	0.08	1.0 (1.0**–**1.0)	0.06
Symptoms of anxiety	2.9 (1.6**–**5.5)	< 0.01	2.2 (1.0**–**4.7)	0.06
Symptoms of depression	2.1 (1.1**–**4.1)	0.03	1.4 (0.6**–**3.2)	0.49
Concerned about the need for TKI treatment in the future	2.1 (1.3**–**3.4)	< 0.01	1.7 (1.1**–**2.9)	**0.03**
Received surgery for the GIST	2.5 (0.9**–**6.7)	0.08	2.6 (0.9**–**7.4)	0.07
**Lacking money to buy basic things as a result of physical condition or medical treatment**	**Univariable logistic regression**		**Multivariable logistic regression** Nagelkerke *R*^2^ = 0.39	
	**OR (95% CI)**	***p*-value**	**OR (95% CI)**	***p*-value**
Current TKI use	2.6 (0.9**–**7.7)	0.08	0.6 (0.1**–**3.0)	0.57
Received surgery for the GIST	0.3 (0.1**–**1.2)	0.08	0.1 (0.0**–**0.8)	**0.03**
Not living with a partner	4.8 (1.6**–**14.3)	< 0.01	7.6 (1.7**–**34.6)	**0.01**
Having ≥ 2 comorbidities	4.4 (1.0**–**20.3)	0.06	2.6 (0.4**–**15.5)	0.31
GIST location other than the stomach	3.3 (1.1**–**10.0)	0.04	3.2 (0.8**–**13.3)	0.11
Declared incapacitated for work	5.4 (1.4**–**20.7)	0.01		
Symptoms of anxiety	10.7 (3.3**–**34.5)	< 0.01	5.0 (0.8**–**31.1)	0.08
Symptoms of depression	9.2 (2.9**–**28.9)	< 0.01	2.2 (0.4**–**12.4)	0.37
Severe fear of recurrence or progression	8.6 (1.9**–**38.9)	< 0.01	3.6 (0.5**–**23.8)	0.19
Concerned about the need for TKI treatment in the future	3.5 (1.0**–**11.5)	0.04	1.3 (0.3**–**5.8)	0.75
Concerned about dying from GIST in the near future	3.0 (1.0**–**9.3)	0.05	0.2 (0.0**–**1.9)	0.18
Concerned about dying from the GIST in the long term future	3.5 (1.0**–**12.7)	0.06	3.7 (0.4**–**31.6)	0.23
**Being in debt as a result of physical condition or medical treatment**	**Univariable logistic regression**		**Multivariable logistic regression** Nagelkerke *R*^2^ = 0.30	
	**OR (95% CI)**	***p*-value**	**OR (95% CI)**	***p*-value**
GIST location other than the stomach	4.5 (0.9**–**23.4)	0.08	2.9 (0.5**–**17.4)	0.24
Symptoms of anxiety	31.7 (3.6**–**277.8)	< 0.01	17.6 (1.4**–**221.9)	**0.03**
Symptoms of depression	14.6 (2.6**–**82.8)	< 0.01	2.5 (0.3**–**19.6)	0.39
**Changing one’s lifestyle because of financial difficulties as a result of physical condition or medical treatment**	**Univariable logistic regression**		**Multivariable logistic regression** Nagelkerke *R*^2^ = 0.52	
	**OR (95% CI)**	***p*-value**	**OR (95% CI)**	***p*-value**
Current TKI use	2.8 (1.3**–**6.1)	0.01	5.2 (0.2**–**115.3)	0.30
Receiving treatment in a palliative setting	2.4 (1.1**–**5.6)	0.04	0.8 (0.0**–**35.6)	0.90
Having ≥ 2 comorbidities	3.7 (1.2**–**11.4)	0.02	2.9 (0.3**–**32.7)	0.40
GIST location other than the stomach	2.1 (1.0**–**4.7)	0.06	0.2 (0.0**–**2.4)	0.18
Less able to work due to GIST and treatment	28.3 (4.7**–**171.7)	< 0.01	31.0 (1.2**–**830.8)	**0.04**
Declared incapacitated for work	4.3 (1.5**–**12.8)	< 0.01		
Symptoms of anxiety	8.3 (3.6**–**19.2)	< 0.01	5.3 (0.2**–**120.8)	0.30
Symptoms of depression	8.6 (3.7**–**20.1)	< 0.01	0.6 (0.0**–**24.0)	0.77
Severe fear of recurrence or progression	5.6 (2.2**–**14.1)	< 0.01	6.8 (0.2**–**215.0)	0.27
Concerned about dying from GIST in the near future	4.6 (2.0**–**10.8)	< 0.01	1.3 (0.1**–**27.6)	0.89
Concerned about dying from the GIST in the long term future	6.7 (2.1**–**18.2)	< 0.01	1.4 (0.0**–**38.7)	0.86
**Having less money to spend on oneself as a result of physical condition or medical treatment**	**Univariable logistic regression**		**Multivariable logistic regression** Nagelkerke *R*^2^ = 0.49	
	**OR (95% CI)**	***p*-value**	**OR (95% CI)**	***p-*value**
Having ≥ 2 comorbidities	2.6 (1.0**–**6.7)	0.05	2.3 (0.3**–**21.5)	0.45
GIST location other than the stomach	2.0 (0.9**–**4.3)	0.08	0.3 (0.0**–**4.2)	0.39
Less able to work due to GIST and treatment	15.9 (2.6**–**96.6)	< 0.01	20.9 (0.9**–**485.0)	0.06
Declared incapacitated for work	4.3 (1.5**–**12.8)	< 0.01		
Symptoms of anxiety	7.7 (3.4**–**17.5)	< 0.01	12.3 (0.3**–**518.3)	0.19
Symptoms of depression	9.6 (4.1**–**22.2)	< 0.01	0.3 (0.0**–**14.0)	0.54
Severe fear of recurrence or progression	4.7 (1.9**–**11.7)	< 0.01	4.1 (0.2**–**104.4)	0.40
Concerned about dying from GIST in the near future	4.1 (1.8**–**9.3)	< 0.01	0.8 (0.0**–**18.1)	0.90
Concerned about dying from the GIST in the long term future	3.1 (1.3**–**7.5)	0.01	1.4 (0.1**–**36.5)	0.84
**Experiencing problems paying regular expenses as a result of physical condition or medical treatment**	**Univariable logistic regression**		**Multivariable logistic regression** Nagelkerke *R*^2^ = 0.24	
	**OR (95% CI)**	***p-*value**	**OR (95% CI)**	***p*-value**
Not living with a partner	2.6 (0.9**–**7.7)	0.09	0.4 (0.1**–**1.5)	0.19
Declared incapacitated for work	11.0 (2.3**–**52.5)	< 0.01		
Symptoms of anxiety	7.6 (2.4**–**23.8)	< 0.01	2.5 (0.5**–**13.2)	0.27
Symptoms of depression	6.5 (2.1**–**20.5)	< 0.01	1.6 (0.3**–**8.2)	0.54
Severe fear of recurrence or progression	18.8 (2.4**–**145.7)	< 0.01	9.7 (1.1**–**89.0)	**0.05**
Concerned about the need for TKI treatment in the future	3.5 (1.0**–**11.5)	0.04	1.4 (0.4**–**5.1)	0.65
Concerned about dying from GIST in the near future	4.3 (1.3**–**14.0)	0.02	0.6 (0.1**–**3.5)	0.61
Concerned about dying from the GIST in the long term future	5.8 (1.3**–**26.2)	0.02	2.6 (0.3**–**21.3)	0.37
**Having to borrow money or sell personal belongings as a result of physical condition or medical treatment**	**Univariable logistic regression**		**Multivariable logistic regression** Nagelkerke *R*^2^ = 0.42	
	**OR (95% CI)**	***p*-value**	**OR (95% CI)**	***p*-value**
Having ≥ 2 comorbidities	6.4 (0.8**–**51.7)	0.08	3.7 (0.4**–**33.0)	0.25
Number of hours working	1.2 (1.0**–**1.4)	0.02		
Symptoms of anxiety	54.4 (6.6**–**446.5)	< 0.01	55.4 (5.4**–**570.0)	** < 0.01**
Symptoms of depression	9.3 (2.4**–**36.3)	< 0.01	0.8 (0.1**–**3.9)	0.75

Data in bold emphasis indicates significant results

### Sensitivity analysis

In the supplementary material, the results of the sensitivity analysis stratified by treatment setting are presented. Similar to patients on current TKI treatment, patients in a palliative treatment setting reported more financial difficulties due to their physical condition or treatment (18.9% vs 8.8%, *p* = 0.01), and more often changed their lifestyle because of financial difficulties (15.6% vs 6.2%, *p* = 0.01) compared to patients treated in a curative setting. In addition, palliative patients had more extra expenses that were difficult to pay (14.4% vs 6.2%, *p* = 0.03), and more often lacked money to buy basic things (8.9% vs 2.7%, *p* = 0.03) compared to patients treated in a curative setting. The percentage of patients that had a paid job was significantly (*p* =  < 0.01) lower in the palliative group (*n* = 15, 17.0%) compared to the curative group (*n* = 84, 37.2%), which was also lower than the 24.3% of patients with a job in the current TKI group. As well as patients not on TKI treatment compared to patients on current TKI treatment, most patients treated in the curative setting did not feel hindered at work, while the majority of patients in the palliative setting did (63.4 vs 26.7, *p* = 0.01). They more frequently reported that working caused them complaints (33.3% vs 9.8%, *p* = 0.03) and felt that they only could work part-time (26.7% vs 6.1%, *p* = 0.03). Regarding patients without jobs, the majority was retired or declared incapacitated for work, with the percentage of patients declared incapacitated for work being higher in the palliative setting (*n* = 18, 24.7%) compared to the curative setting (*n* = 7, 4.9%). The logistic regression analysis showed similar patterns, except that having symptoms of anxiety was no longer associated with having extra expenses that were difficult to pay.

### Impact of financial difficulties on HRQoL

**Fig. 1:**
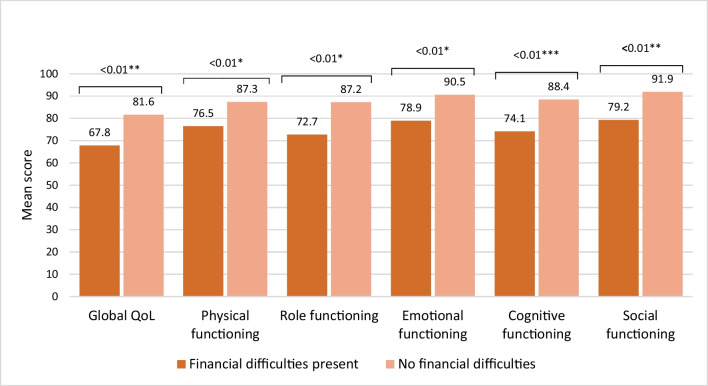
Comparison of mean scores on global QoL and functioning scales of the EORTC QLQ-C30 among GIST patients (*n* = 325) with financial difficulties and without financial difficulties. On global QoL and functioning scales, higher scores indicate a better global quality of life and functioning. The mean differences between both groups were considered *small, **medium or ***large [31]

As is shown in Fig. 1, having financial difficulties significantly reduced global quality of life, as well as physical, role, emotional, cognitive and social functioning as compared to patients who did not report financial difficulties.

## Discussion

Financial difficulties are an increasing topic of interest in cancer care, and our study is the largest cohort of GIST patients, until now, in which the prevalence of financial difficulties and the impact on patients’ well-being were studied. We assessed financial difficulties using the nine financial toxicity-related items of the EORTC item library [21], which gave us the opportunity to assess a broader range of financial difficulties, eminently when compared to studies which only used the single question of the EORTC QLQ-C30 [28]. Within our GIST population, the most frequently reported financial difficulties were having extra expenses (35.4%), followed by financial difficulties caused by their physical condition or treatment (11.7%). Four out of nine items (i.e. being in debt, having to borrow money or sell personal belongings, problems with paying regular expenses, lacking money to buy basic things) were barely (< 5%) reported by GIST patients.

Within a healthy Dutch norm population, aged 60–69 years, the mean financial difficulty score was 4.7 (range 0–100) [29]. When comparing this to our data, both patients on TKIs (mean 8.8) and those treated in a palliative setting (mean 9.3) had a higher mean score, albeit the mean differences are considered small differences [30]. Patients not on TKIs (mean 4.2) and in a curative treatment setting (mean 4.4) had slightly lower scores. This suggests that patients on TKIs, certainly those in a palliative setting, are more likely to report financial difficulties. Although the prevalence of financial difficulties caused by the physical condition or treatment was significantly higher in GIST patients on current TKIs (17.3%) or in a palliative treatment setting (18.9%) compared to patients not on TKIs (8.7%) or in a curative setting (8.8%), this was still substantially lower compared to sarcoma, lung, breast or ovarian cancer patients and cancer patients undergoing radiotherapy, with 26–45% reporting financial difficulties [12, 31, 32]. Previous research identified younger age at diagnosis, female sex, advanced or recurrent cancer, receiving anticancer drugs or radiation treatment, a low income and unemployment as potential risk factors for developing financial difficulties [33–36]. Therefore, the low prevalence in our study might be explained by the fact that our study population was older, the majority did not have a job due to retirement, and there were a relatively low number of patients on active treatment and in a palliative treatment setting. Furthermore, in the Netherlands, the costs of most healthcare, including the cost of TKIs and follow-up care, are covered by healthcare insurance. This leads to less healthcare costs for patients; subsequently, patients may experience fewer financial difficulties. Besides that, we have a good pension scheme in the Netherlands, which is relevant given the mean age in our study and the fact that a large proportion was retired, which could have attributed to the lower prevalence of financial difficulties as well.

It could be hypothesized that patients who do not receive active treatment because they have completed their curative treatment are better able to live their lives without limitations and have work in comparison to patients on active TKI treatment, especially those in a palliative setting. This was supported by our data as the percentage of GIST patients without a job was significantly higher in patients on current TKIs, and those treated with a palliative intent, and generally due to being declared incapacitated for work. Furthermore, of the patients with a job while on current TKIs or in a palliative setting, approximately half and three-quarters, respectively, indicated that they experienced limitations in their work. Additionally, our logistic regression analysis showed that being less able to work was associated with higher odds of experiencing financial difficulties due to the GIST or its treatment.

In line with previous research among cancer patients [31, 37], experiencing financial difficulties had a considerable impact on the GIST patients’ HRQoL; they had a significant lower global quality of life and worse functioning on all scales in comparison to patients who did not report financial difficulties. In addition, consistent with other studies [38, 39], financial difficulties were associated with psychological symptoms, possibly explaining the lower emotional functioning. Previous studies [40, 41] in GIST patients already reported that patients with severe fear of cancer recurrence or progression experienced more psychological distress and had a poorer psychological well-being; they also experienced more limitations in work, daily and social activities than did patients who experienced less fear. This leads us to the question, a chicken and the egg dilemma; are patients burdened by their financial difficulties causing them to experience fear and psychological distress, or, are their fear and psychological distress the reasons that they function less well and get into financial problems?

To the best of our knowledge, this is the largest cohort of GIST patients in which financial difficulties were studied. Our study is a diverse representation of the clinical practice, as patients were in follow-up not receiving active treatment, and others were receiving TKIs in either a curative or palliative setting. In addition, we used the nine items of the EORTC item library to assess a broader range of financial difficulties, as one of the first, rather than the single question of the EORTC QLQ-C30. Our study also has some limitations. First, the cross-sectional design of this study did not allow us to investigate changes in financial difficulties over time and possible causalities. Second, this multicentre study was conducted in the Netherlands; therefore, only Dutch GIST patients were included, which could impede the generalizability. As mentioned earlier, the Dutch healthcare system differs from other countries and most healthcare, including the costs of TKIs, is covered by a mandatory healthcare insurance. Of note, in the Netherlands, no options exist to pay for TKIs out-of-pocket when the healthcare insurance companies do not reimburse them because of the national regulations, which is the case for ripretinib currently. Besides the broad health insurance coverage, we also have a good pension scheme in the Netherlands. Both factors may have attributed to the lower prevalence of financial difficulties compared to other countries. Third, our study is not representative for the entire Dutch GIST population as illiterate and low-literate patients probably did not participate, while these patients most likely have more financial difficulties. Last, we did not collect income data of patients, and could not take this into account in our analysis.

Attention to the possible presence of financial difficulties is increasingly important, given that healthcare and treatments, but also costs of living in general, in an ageing population are becoming more expensive over the years. Hence, it is to be expected that the occurrence of financial difficulties will increase, also in the Netherlands. Financial difficulties are not much GIST specific, but can occur among all types of (cancer) patients [42]. Awareness and identifying financial difficulties are not primarily a matter for oncologists, but can be addressed by the social workers in the team, and are in fact topics that policy makers should incorporate in their future on socio-economic strategies as well [43]. Whether or not patients get into financial difficulties due to the cancer and treatment can differ per country, and if patients are depending on TKIs and have to pay for this high-cost treatment themselves, this can potentially be an enormous burden.

## Conclusion

In conclusion, even in a country where the costs of TKIs and follow-up care are covered by healthcare insurance, financial difficulties can be present in GIST patients, especially in patients on TKI treatment and in a palliative treatment setting.

### Supplementary Information

Below is the link to the electronic supplementary material.

Supplementary file1 (DOCX 41 KB)

## Data Availability

Data are available on reasonable request from the corresponding author.
