# Supplementary material for: Financial difficulties experienced by patients with gastrointestinal stromal tumours (GIST) in the Netherlands: data from a cross-sectional multicentre study
Source: Support Care Cancer. 2024 Apr 10;32(5):279. doi: 10.1007/s00520-024-08451-0 (PMC11004045; doi:10.1007/s00520-024-08451-0)
Supplement: Supplementary file 1 — Supplementary file1 (DOCX 41 KB) [file 520_2024_8451_MOESM1_ESM.docx]

Supplementary table 1. Sensitivity analysis - Patient characteristics stratified by treatment setting based on data of the DGR

|  | **Total**  **(n=328)** | **Curative setting (n=235)** | **Palliative setting (n=93)** | ***p*-value** |
| --- | --- | --- | --- | --- |
| Sex *n (%)*  Male  Female | 174 (53.0)  154 (47.0) | 121 (51.5)  114 (48.5) | 53 (57.0)  40 (43.0) | 0.37 |
| Age at survey completion *Mean ± SD* | 66.7 ± 10.4 | 66.2 ± 10.8 | 68.0 ± 9.2 | 0.15 |
| Socio-economic status *n (%)*  Low  High | 150 (45.7)  178 (54.3) | 100 (42.6)  135 (57.4) | 50 (53.8)  43 (46.2) | 0.07 |
| Marital stage *n (%)*  Married / Living with partner  Not living with a partner  Missing | 246 (75.7)  79 (24.3)  3 | 174 (75.0)  58 (25.0)  3 | 72 (77.4)  21 (22.6)  - | 0.65 |
| Educational level* *n (%)*  Low/intermediate  High  Missing | 206 (64.0)  116 (36.0)  6 | 145 (63.3)  84 (36.7)  6 | 61 (65.6)  32 (34.4)  - | 0.70 |
| Comorbidity *n (%)*  None  1  ≥2  Missing | 109 (33.4)  71 (21.8)  146 (44.8)  2 | 82 (35.0)  50 (21.4)  102 (43.6)  1 | 27 (29.3)  21 (22.8)  44 (47.8)  1 | 0.62 |
| Time since diagnosis in years *Mean ± SD* | 5.9 ± 2.8 | 5.7 ± 2.8 | 6.3 ± 2.8 | 0.06 |
| Location primary GIST *n (%)*  Stomach  Small intestine  Rectum  Other | 207 (63.1)  79 (24.1)  21 (6.4)  21 (6.4) | 165 (70.2)  48 (20.4)  14 (6.0)  8 (3.4) | 42 (45.2)  31 (33.3)  7 (7.5)  13 (14.0) | **<0.01** ͣ |
| Received TKI at some point  Neo-adjuvant  Adjuvant  Neo-adjuvant and adjuvant  Palliative | 219 (66.8)  39 (17.8)  45 (20.5)  42 (19.2)  93 (42.5) | 126 (53.6)  39 (31.0)  45 (35.7)  42 (33.3)  - | 93 (100.0)  -  -  -  93 (100%) | **<0.01** |
| Current TKI | 116 (35.4) | 27 (11.5) | 89 (95.7) | **<0.01** |
| Received previous surgery for the GIST  Yes  No | 300 (91.5)  28 (8.5) | 232 (98.7))  3 (1.3) | 68 (73.1)  25 (26.9) | **<0.01** ͣ |
| Phase of treatment and follow up  Declared cured, no follow-up  Not receiving active treatment, in follow up  Active treatment with curative intent  Treated with palliative intent | 61 (18.6)  147 (44.8)  27 (8.2)  93 (28.4) | 61 (26.0)  147 (62.6)  27 (11.5)  - | -  -  -  93 (100.0) | **<0.01** ͣ |
| Abbreviations: TKI = Tyrosine Kinase Inhibitor, SD = standard deviation  *Low (primary and secondary education), intermediate ((secondary) vocational education), and high (higher vocational education and academic education) educational level  ͣ Fisher’s exact test or likelihood ratio | | | | |

Supplementary table 2. Sensitivity analysis - Patient reported financial difficulties as a result of the GIST or medical treatment of patients in a curative and palliative setting

|  |  | **Total**  **(n=316*)** | **Curative setting (n=226)** | **Palliative setting (n=90)** | ***p*-value** |
| --- | --- | --- | --- | --- | --- |
| Physical condition or treatment causing financial difficulties | Yes  No | 37 (11.7)  279 (88.3) | 20 (8.8)  206 (91.2) | 17 (18.9)  73 (81.1) | **0.01** |
|  | Mean ± SD | 5.8 ± 17.8 | 4.4 ± 15.7 | 9.3 ± 21.8 | 0.06 |
| Having extra expenses that were difficult to pay | Yes  No | 27 (8.5)  289 (91.5) | 14 (6.2)  212 (93.8) | 13 (14.4)  77 (85.6) | **0.03** ͣ |
|  | Mean ± SD | 3.3 ± 11.3 | 2.4 ± 9.7 | 5.6 ± 14.4 | 0.05 |
| Having extra expenses | Yes  No | 112 (35.4)  204 (64.6) | 75 (33.2)  151(66.8) | 37 (41.1)  53 (58.9) | 0.18 |
|  | Mean ± SD | 15.5 ± 23.0 | 14.5 ± 22.4 | 18.1 ± 24.6 | 0.20 |
| Lacking money to buy basic things | Yes  No | 14 (4.4)  302 (95.6) | 6 (2.7)  220 (97.3) | 8 (8.9)  82 (91.1) | **0.03** ͣ |
|  | Mean ± SD | 2.1 ± 10.4 | 1.2 ± 7.6 | 4.4 ± 15.2 | 0.05 |
| Being in debt | Yes  No | 7 (2.2)  309 (97.8) | 3 (1.3)  223 (98.7) | 4 (4.4)  86 (95.6) | 0.11 ͣ |
|  | Mean ± SD | 1.1 ± 7.4 | 0.4 ± 3.8 | 2.6 ± 12.5 | 0.11 |
| Changing one's lifestyle because of financial difficulties | Yes  No | 28 (8.9)  288 (91.1) | 14 (6.2)  212 (93.8) | 14 (15.6)  76 (84.4) | **0.01** ͣ |
|  | Mean ± SD | 4.6 ± 16.1 | 3.4 ± 14.2 | 7.8 ± 20.0 | 0.06 |
| Having less money to spend on oneself | Yes  No | 29 (9.2)  287 (90.8) | 17 (7.5)  209 (92.5) | 12 (13.3)  78 (86.7) | 0.13 ͣ |
|  | Mean ± SD | 4.6 ± 15.9 | 3.5 ± 13.2 | 7.4 ± 21.1 | 0.11 |
| Experiencing problems paying regular expenses | Yes  No | 14 (4.4)  302 (95.6) | 8 (3.5)  218 (96.5) | 6 (6.7)  84 (93.3) | 0.23 ͣ |
|  | Mean ± SD | 2.0 ± 9.9 | 1.5 ± 8.2 | 3.3 ± 13.3 | 0.22 |
| Having to borrow money or sell personal belongings | Yes  No | 9 (2.9)  306 (97.1) | 5 (2.2)  220 (97.8) | 4 (4.4)  86 (95.6) | 0.28 ͣ |
|  | Mean ± SD | 1.2 ± 7.2 | 0.9 ± 6.2 | 1.9 ± 9.2 | 0.36 |
| *9 patients did not completed the items regarding financial difficulties  ͣ Fisher’s exact test | | | | | |

Supplementary table 3. Sensitivity analysis - Patient reported work-related information of GIST patients in a curative and palliative setting

| **Patients with a paid job** | **Total**  **(n=99*)** | **Curative setting (n=84*)** | **Palliative setting (n=15)** | ***p*-value** |
| --- | --- | --- | --- | --- |
| Working hours**  Mean ± SD  Range | 32.1 ± 10.8  6 – 60 | 31.9 ± 10.5  6 – 60 | 33.0 ± 12.3  15 – 60 | 0.71 |
| Type of work  Physical  Mental  Both physical and mental | 8 (8.2)  54 (55.7)  35 (36.1) | 6 (7.3)  45 (54.9)  31 (37.8) | 2 (13.3)  9 (60.0)  4 (26.7) | 0.60 ͣ |
| Able to work  Not – less able  Good – excellent | 7 (7.2%)  90 (92.8%) | 4 (4.9)  78 (95.1) | 3 (20.0)  12 (80.0) | 0.07 ͣ |
| Work is hindered by  Nothing  Working causes me some complaints  Have to reduce my work pace or adjust my way of working  Often have to reduce my work pace or adjust my way of working  Have the feeling that I can only work part-time  I dare not to apply for another job  I am not able to work at all | 56 (57.7)  13 (13.4)  21 (21.6)  10 (10.3)  9 (9.3)  5 (5.2)  2 (2.1) | 52 (63.4)  8 (9.8)  16 (19.5)  8 (9.8)  5 (6.1)  3 (3.7)  2 (2.4) | 4 (26.7)  5 (33.3)  5 (33.3)  2 (13.3)  4 (26.7)  2 (13.3)  0 (0.0) | **0.01** ͣ  **0.03** ͣ  0.31 ͣ  0.65 ͣ  **0.03** ͣ  0.17 ͣ  1.00 ͣ |
| **Patients not having a paid job** | **Total**  **(n=215)** | **Curative setting (n=142)** | **Palliative setting (n=73)** | ***p*-value** |
| Reasons for not having a paid job  Retired  Unwillingly without a job  Declared incapacitated  Staying home wife/man, caregiver | 178 (82.8)  5 (2.3)  25 (11.6)  7 (3.3) | 128 (90.1)  4 (2.8)  7 (4.9)  3 (2.1) | 50 (68.5)  1 (1.4)  18 (24.7)  4 (5.5) | **<0.01** ͣ |
| Declared incapacitated due to cancer  Yes  Missing | 22 (91.7)  1 | 5 (71.4)  - | 17 (100%)  1 | 0.08 ͣ |
| Declared incapacitated, for  70-80%  100%  Missing | 5  18  2 | 2  3  2 | 3  15  - |  |
| *Two missing, these two patients indicated having a job but did not completed the work-related questions.  ** In the Netherlands an average of 36 working hours is considered a full-time job  ͣ Fisher’s exact test | | | | |

Supplementary table 4. Sensitivity analysis - Logistic regression models evaluating factors associated with various financial difficulties

| **Physical condition or treatment causing financial difficulties** | **Univariable logistic regression** | | **Multivariable logistic regression**  Nagelkerke *R²* = 0.35 | |
| --- | --- | --- | --- | --- |
|  | **OR (95% CI)** | ***p*-value** | **OR (95% CI)** | ***p*-value** |
| Current TKI use | 2.6 (1.3 – 5.3) | 0.01 | 4.8 (0.8-27.2) | 0.07 |
| Treated in a palliative setting | 2.4 (1.2 – 4.8) | 0.01 | 1.0 (0.1-10.8) | 0.98 |
| Time since GIST diagnosis (in years) | 1.1 (1.0-1.3) | 0.07 | 1.2 (0.9-1.5) | 0.21 |
| GIST location other than stomach | 2.9 (1.4-5.8) | <0.01 | 0.9 (0.2-4.7) | 0.90 |
| Less able to work due to GIST and treatment | 13.5 (2.6-71.3) | <0.01 | 14.9 (1.4-160.4) | **0.03** |
| Declared incapacitated for work | 4.2 (1.5-11.6) | 0.01 |  |  |
| Symptoms of anxiety | 5.5 (2.6-11.7) | <0.01 | 7.5 (0.7-80.3) | 0.10 |
| Symptoms of depression | 5.0 (2.3-11.1) | <0.01 | 0.1 (0.0-2.7) | 0.17 |
| Severe fear of recurrence or progression | 4.1 (1.9-9.0) | <0.01 | 3.0 (0.4-21.3) | 0.27 |
| Concerned about the need for TKI treatment in the future | 2.0 (1.0-4.0) | 0.06 | 2.1 (0.4-12.4) | 0.40 |
| Concerned about dying from GIST in the near future | 3.4 (1.7-7.1) | <0.01 | 1.2 (0.1-10.2) | 0.87 |
| Concerned about dying from the GIST in the long term future | 3.2 (1.4-7.02) | <0.01 | 1.1 (0.1-10.0) | 0.96 |
| **Having extra expenses that were difficult to pay as a result of physical condition or medical treatment** | **Univariable logistic regression** | | **Multivariable logistic regression**  Nagelkerke R² = 0.43 | |
|  | **OR (95% CI)** | ***p*-value** | **OR (95% CI)** | ***p*-value** |
| Not living with a partner | 2.1 (.9-4.8) | 0.08 | 1.8 (0.2-15.6) | 0.58 |
| Current TKI use | 2.0 (0.9 – 4.5) | 0.08 | 0.1 (0.0-6.2) | 0.31 |
| Treated in a palliative setting | 2.6 (1.2 – 5.7) | 0.02 | 2.7 (0.0-216.0) | 0.65 |
| Having ≥2 comorbidities | 3.0 (1.1-8.2) | 0.04 | 1.7 (0.2-14.5) | 0.62 |
| Less able to work due to GIST and treatment | 4.7 (0.8-28.7) | 0.10 | 13.4 (0.7 – 264.8) | 0.09 |
| Declared incapacitated for work | 3.8 (1.2-11.9) | 0.03 |  |  |
| Symptoms of anxiety | 7.5 (3.2-17.4) | <0.01 | 24.8 (0.9-678.6) | 0.05 |
| Symptoms of depression | 6.3 (2.7-15.0) | <0.01 | 0.0 (0.0-2.9) | 0.14 |
| Severe fear of recurrence or progression | 5.3 (2.1-13.4) | <0.01 | 6.6 (0.5-87.5) | 0.15 |
| Concerned about the need for TKI treatment in the future | 2.5 (1.1-5.8) | 0.03 | 4.9 (0.5-45.5) | 0.16 |
| Concerned about dying from GIST in the near future | 4.3 (1.8-10.2) | <0.01 | 0.4 (0.0-4.2) | 0.44 |
| Concerned about dying from the GIST in the long term future | 5.9 (2.0-17.4) | <0.01 | 2.2 (0.2-30.0) | 0.56 |
| **Having extra expenses as a result of physical condition or medical treatment** | **Univariable logistic regression** | | **Multivariable logistic regression**  Nagelkerke R² = 0.19 | |
|  | **OR (95% CI)** | ***p*-value** | **OR (95% CI)** | ***p*-value** |
| Age at moment of questionnaire (in years) | 1.0 (1.0-1.0) | 0.08 | 1.0 (1.0-1.0) | 0.08 |
| Symptoms of anxiety | 2.9 (1.6-5.5) | <0.01 | 2.2 (1.0-4.8) | 0.06 |
| Symptoms of depression | 2.1 (1.1-4.1) | 0.03 | 1.3 (0.6-3.1) | 0.49 |
| Concerned about the need for TKI treatment in the future | 2.1 (1.3-3.4) | <0.01 | 1.8 (1.1-2.9) | **0.03** |
| Received surgery for the GIST | 2.5 (0.9-6.7) | 0.08 | 2.4 (0.9-6.9) | 0.10 |
| **Lacking money to buy basic things as a result of physical condition or medical treatment** | **Univariable logistic regression** | | **Multivariable logistic regression**  Nagelkerke R² = 0.39 | |
|  | **OR (95% CI)** | ***p*-value** | **OR (95% CI)** | ***p*-value** |
| Treated in a palliative setting | 3.6 (1.2 – 10.6) | 0.02 | 0.8 (0.2-3.9) | 0.77 |
| Received surgery for the GIST | 0.3 (0.1-1.2) | 0.08 | 0.1 (0.0-0.9) | **0.04** |
| Not living with a partner | 4.8 (1.6-14.3) | <0.01 | 6.5 (1.5-27.5) | **0.01** |
| Having ≥2 comorbidities | 4.4 (1.0-20.3) | 0.06 | 2.6 (0.4-15.0) | 0.30 |
| GIST location other than stomach | 3.3 (1.1-10.0) | 0.04 | 2.8 (0.6-11.8) | 0.15 |
| Declared incapacitated for work | 4.9 (1.3-18.1) | 0.02 |  |  |
| Symptoms of anxiety | 10.7 (3.3-34.5) | <0.01 | 4.8 (0.8-29.1) | 0.09 |
| Symptoms of depression | 9.2 (2.9-28.9) | <0.01 | 2.2 (0.4-12.5) | 0.37 |
| Severe fear of recurrence or progression | 8.6 (1.9-38.9) | <0.01 | 4.2 (0.6-28.9) | 0.15 |
| Concerned about the need for TKI treatment in the future | 3.5 (1.0-11.5) | 0.04 | 1.3 (0.3-6.0) | 0.71 |
| Concerned about dying from GIST in the near future | 3.0 (1.0-9.3) | 0.05 | 0.3 (0.0-2.0) | 0.19 |
| Concerned about dying from the GIST in the long term future | 3.5 (1.0-12.7) | 0.06 | 4.0 (0.4-35.6) | 0.22 |
| **Being in debt as a result of physical condition or medical treatment** | **Univariable logistic regression** | | **Multivariable logistic regression**  Nagelkerke R² = 0.30 | |
|  | **OR (95% CI)** | ***p*-value** | **OR (95% CI)** | ***p*-value** |
| GIST location other than stomach | 4.5 (0.9-23.4) | 0.08 | 2.9 (0.5-17.4) | 0.24 |
| Symptoms of anxiety | 31.7 (3.6-277.8) | <0.01 | 17.6 (1.4-221.9) | **0.03** |
| Symptoms of depression | 14.6 (2.6-82.8) | <0.01 | 2.5 (0.3-19.6) | 0.39 |
| **Changing one's lifestyle because of financial difficulties as a result of physical condition or medical treatment** | **Univariable logistic regression** | | **Multivariable logistic regression**  Nagelkerke R² = 0.57 | |
|  | **OR (95% CI)** | ***p*-value** | **OR (95% CI)** | ***p*-value** |
| Current TKI use | 2.6 (1.2-5.7) | 0.02 | 3.9 (0.3-50.7) | 0.29 |
| Treated in a palliative setting | 2.8 (1.3-6.1) | 0.01 | 0.9 (0.0-25.7) | 0.96 |
| Having ≥2 comorbidities | 3.7 (1.2-11.4) | 0.02 | 3.2 (0.3-35.8) | 0.34 |
| GIST location other than stomach | 2.1 (1.0-4.7) | 0.06 | 0.2 (0.0-2.6) | 0.20 |
| Less able to work due to GIST and treatment | 28.3 (4.7-171.7) | <0.01 | 31.6 (1.2-826.7) | **0.04** |
| Declared incapacitated for work | 4.2 (1.4-12.5) | <0.01 |  |  |
| Symptoms of anxiety | 8.3 (3.6-19.2) | <0.01 | 5.2 (0.2-112.4) | 0.30 |
| Symptoms of depression | 8.6 (3.7-20.1) | <0.01 | 0.4 (0.0-28.2) | 0.83 |
| Severe fear of recurrence or progression | 5.6 (2.2-14.1) | <0.01 | 6.4 (0.2-189.4) | 0.28 |
| Concerned about dying from GIST in the near future | 4.6 (2.0-10.8) | <0.01 | 1.1 (0.1-23.2) | 0.94 |
| Concerned about dying from the GIST in the long term future | 6.7 (2.1-18.2) | <0.01 | 1.3 (0.0-33.6) | 0.89 |
| **Having less money to spend on oneself as a result of physical condition or medical treatment** | **Univariable logistic regression** | | **Multivariable logistic regression**  Nagelkerke R² = 0.47 | |
|  | **OR (95% CI)** | ***p*-value** | **OR (95% CI)** | ***p-*value** |
| Having ≥2 comorbidities | 2.6 (1.0-6.7) | 0.05 | 2.3 (0.3-21.5) | 0.45 |
| GIST location other than stomach | 2.0 (0.9-4.3) | 0.08 | 0.3 (0.0-4.2) | 0.39 |
| Less able to work due to GIST and treatment | 15.9 (2.6-96.6) | <0.01 | 20.9 (0.9-485.0) | 0.06 |
| Declared incapacitated for work | 3.9 (1.3-11.3) | <0.01 |  |  |
| Symptoms of anxiety | 7.7 (3.4-17.5) | <0.01 | 12.3 (0.3-518.3) | 0.19 |
| Symptoms of depression | 9.6 (4.1-22.2) | <0.01 | 0.3 (0.0-14.0) | 0.54 |
| Severe fear of recurrence or progression | 4.7 (1.9-11.7) | <0.01 | 4.1 (0.2-104.4) | 0.40 |
| Concerned about dying from GIST in the near future | 4.1 (1.8-9.3) | <0.01 | 0.8 (0.0-18.1) | 0.90 |
| Concerned about dying from the GIST in the long term future | 3.1 (1.3-7.5) | 0.01 | 1.4 (0.1-36.5) | 0.84 |
| **Experiencing problems paying regular expenses as a result of physical condition or medical treatment** | **Univariable logistic regression** | | **Multivariable logistic regression**  Nagelkerke R² = 0.24 | |
|  | **OR (95% CI)** | ***p-*value** | **OR (95% CI)** | ***p*-value** |
| Not living with a partner | 2.6 (0.9-7.7) | 0.09 | 2.4 (0.7-8.7) | 0.19 |
| Declared incapacitated for work | 8.7 (2.0-37.4) | <0.01 |  |  |
| Symptoms of anxiety | 7.6 (2.4-23.8) | <0.01 | 2.5 (0.5-13.2) | 0.27 |
| Symptoms of depression | 6.5 (2.1-20.5) | <0.01 | 1.6 (0.3-8.2) | 0.54 |
| Severe fear of recurrence or progression | 18.8 (2.4-145.7) | <0.01 | 9.7 (1.1-89.0) | **0.05** |
| Concerned about the need for TKI treatment in the future | 3.5 (1.0-11.5) | 0.04 | 1.4 (0.4-5.1) | 0.65 |
| Concerned about dying from GIST in the near future | 4.3 (1.3-14.0) | 0.02 | 0.6 (0.1-3.5) | 0.61 |
| Concerned about dying from the GIST in the long term future | 5.8 (1.3-26.2) | 0.02 | 2.6 (0.3-21.3) | 0.37 |
| **Having to borrow money or sell personal belongings as a result of physical condition or medical treatment** | **Univariable logistic regression** | | **Multivariable logistic regression**  Nagelkerke R² = 0.42 | |
|  | **OR (95% CI)** | ***p*-value** | **OR (95% CI)** | ***p*-value** |
| Having ≥2 comorbidities | 6.4 (0.8-51.7) | 0.08 | 3.7 (0.4-33.0) | 0.25 |
| Number of hours working | 1.2 (1.0-1.4) | 0.02 |  |  |
| Symptoms of anxiety | 54.4 (6.6-446.5) | <0.01 | 55.4 (5.4-570.0) | **<0.01** |
| Symptoms of depression | 9.3 (2.4-36.3) | <0.01 | 0.8 (0.1-3.9) | 0.75 |
